# Prognostic Utility of High-Sensitivity Cardiac Troponin I in Hemodialysis Patients Without Acute Coronary Syndrome: A Retrospective Cohort Study

**DOI:** 10.7759/cureus.86125

**Published:** 2025-06-16

**Authors:** Sara Serrano-Leal, Luis Ruiz-Peña, Fabian Jaimes, Joaquin Rodelo-Ceballos

**Affiliations:** 1 Internal Medicine, Universidad de Antioquia, Medellin, COL; 2 Internal Medicine, Universidad de Antioquia, Medellín, COL; 3 Nephrology, Nephrology Unit, San Vicente Fundation University Hospital, Medellín, COL

**Keywords:** all-cause mortality, end-stage chronic kidney disease, hemodialysis, high-sensitivity troponin i, cardiovascular diseases

## Abstract

Background and objectives

In patients with end-stage kidney disease (ESKD) undergoing hemodialysis (HD), high-sensitivity cardiac troponin T (hs-cTnT) is a well-established biomarker for predicting all-cause and cardiovascular mortality. However, the prognostic utility of high-sensitivity cardiac troponin I (hs-cTnI) in this population remains uncertain. This study aimed to evaluate the association between hs-cTnI levels and one-year all-cause mortality in ESKD patients on HD presenting without suspected acute coronary syndrome (ACS).

Materials and methods

We performed a retrospective cohort study including 236 ESKD patients on HD admitted to a tertiary care hospital. hs-cTnI was measured within 24 hours of admission in the absence of clinical suspicion of ACS. Demographic, clinical, and laboratory data were collected. The primary outcome was all-cause mortality at one year. Multivariate logistic regression was used to assess the independent association between elevated hs-cTnI (above the 99th percentile) and mortality.

Results

A total of 236 patients were included. Elevated hs-cTnI was found in 133 (56.4%) patients, while 103 (43.6%) had non-elevated levels. One-year mortality was higher in the elevated group (43.6% [58/133]) than in the non-elevated group (27.1% [28/103]). However, after adjustment for potential confounders, elevated hs-cTnI was not independently associated with one-year mortality (adjusted odds ratio [aOR]: 1.73; 95% confidence interval [CI]: 0.97-3.08).

Conclusions

In ESKD patients on HD without suspected ACS, hs-cTnI measured within 24 hours of admission was not an independent predictor of one-year all-cause mortality. These findings suggest limited prognostic utility of hs-cTnI in this setting and support further investigation into the potential superiority of hs-cTnT in this population.

## Introduction

Chronic kidney disease (CKD) is associated with increased mortality from both cardiovascular and non-cardiovascular causes. In patients with end-stage kidney disease (ESKD) on hemodialysis (HD), the estimated three-year mortality rate is approximately 39% [1]. Cardiovascular disease accounts for up to 50% of deaths in this population [2,3]. However, non-cardiovascular causes are also highly prevalent and may even surpass cardiovascular mortality [1].

To better stratify mortality risk in this population, several biomarkers have been proposed, commonly classified as inflammatory, nutritional, or cardiovascular. Inflammatory biomarkers include tumor necrosis factor-alpha (TNF-α), C-reactive protein (CRP), and interleukin-6 (IL-6); nutritional markers include albumin and prealbumin; and cardiovascular markers include B-type natriuretic peptide (BNP), N-terminal pro-B-type natriuretic peptide (NT-proBNP), and cardiac troponins, with the latter being the most frequently associated with both cardiovascular and all-cause mortality [4,5].

Cardiac troponin I (cTnI) and troponin T (cTnT) are structural proteins specific to myocardial cells and are widely used as biomarkers of myocardial ischemia. Compared to cTnI, cTnT has a longer half-life, is more readily cleared by the kidneys, and is not removed by dialysis membranes [6,7]. Troponin levels are frequently elevated in patients with kidney failure even in the absence of myocardial ischemia [8]. Among CKD patients, approximately 57% show elevated cTnT levels, while only 17% show elevated cTnI levels [9]. Elevated troponin levels have been independently associated with increased cardiovascular and all-cause mortality in patients with CKD stages 3-5 [10].

Most evidence on the prognostic value of troponins in dialysis patients stems from studies conducted in the early 2000s using conventional assays. These studies demonstrated a strong association between elevated cTnT and cardiovascular mortality, with follow-up ranging from 12 to 24 months [11-15]. However, with the introduction of high-sensitivity troponin assays, especially high-sensitivity cardiac troponin I (hs-cTnI), only a few studies have been published, and their findings have been inconsistent [16-19].

Given this uncertainty and the fact that hs-cTnI is the only troponin assay available in many settings, we aimed to evaluate the prognostic significance of hs-cTnI measured within the first 24 hours of hospitalization in ESKD patients on HD without clinical suspicion of acute coronary syndrome (ACS). The primary outcome was one-year all-cause mortality.

## Materials and methods

Study design

We conducted a retrospective cohort study with a one-year follow-up, including ESKD patients on maintenance HD who had hs-cTnI levels measured between January 2016 and January 2023.

Study site

The study was performed at Hospital San Vicente Fundación in Medellín, Colombia. It received approval from the Institutional Ethics Committee and the Research Committee of the Department of Internal Medicine at the University of Antioquia. The study complied with the ethical standards established by Resolution 8430 of 1993 from the Colombian Ministry of Health.

Participants

We included patients aged ≥18 years with a diagnosis of ESKD on HD for at least three months who had hs-cTnI measured within the first 24 hours of admission to the emergency department. Patients were excluded if they had clinical suspicion of ACS (defined as chest pain suggestive of ischemia, electrocardiographic changes indicative of myocardial injury, or a documented suspicion of ACS in the medical record), suspected or confirmed SARS-CoV-2 infection, peritoneal dialysis, active oncologic disease, or referral from another institution with a prior hospitalization exceeding 24 hours.

Variables

The exposure variable was hs-cTnI, categorized as elevated when exceeding the 99th percentile (P99) of the respective assay. The primary outcome was one-year all-cause mortality. Secondary outcomes included three-month rehospitalization and cardiovascular mortality (Appendix A). Based on literature review and data availability, we selected the following potential confounders: age, history of smoking, diabetes mellitus, and chronic heart failure [1-6,18], as documented in medical records.

Data measurement

hs-cTnI levels were measured using two different assays, depending on the time period. From 2016 to 2017, the Abbott® Architect assay was used, which has a detection limit of 1.9 ng/L and a P99 reference value of 26.2 ng/L. From 2018 onward, the Siemens® Atellica™ assay was implemented, with a detection limit of 1.2 ng/L and a P99 value of 45 ng/L. All samples were processed in the hospital’s central laboratory, which conducts all institutional testing. The laboratory follows an internal quality control program for each assay and is subject to external quality monitoring by both national and international regulatory bodies.

Outcome assessment

All-cause mortality, cardiovascular mortality, and relevant demographic, clinical, and laboratory data were obtained from electronic medical records, the national health system registry (ADRES), and telephone follow-up using contact information from hospital records.

Bias control

Given the high prevalence of comorbidities in the study population, several potential confounders were identified that could influence mortality outcomes. To address this, an adjusted multivariate analysis was conducted to control for these confounding factors (see variable definitions).

Sample size

To estimate the sample size, a 1:2 ratio was expected between ESKD patients with positive hs-cTnI and those with negative hs-cTnI values. Additionally, one-year mortality was estimated at 25% for patients with a positive test result and 15% for those with a negative result. With 80% power and an alpha error of 0.05, the estimated sample size was 207 patients with positive hs-cTnI values and 414 with negative hs-cTnI values, for a total of 621 patients.

Statistical analysis

Qualitative variables were presented as absolute and relative frequencies, while quantitative variables were reported as means with standard deviations or medians with interquartile ranges, depending on normality distribution. A multivariate logistic regression analysis was performed to assess the association between hs-cTnI levels and the primary and secondary outcomes (all-cause mortality, cardiovascular mortality, and rehospitalization at three months), adjusting for age, history of smoking, diabetes mellitus, and chronic heart failure. Results were reported as odds ratios (OR) with 95% confidence intervals (CI).

For missing data, the multivariate analysis was restricted to patients with complete data on adjustment variables. Statistical analysis was performed using STATA-14 (licensed for the University of Antioquia).

Research involving human participants and/or animals

The study protocol was reviewed and approved by the Ethics Committee of the Hospital San Vicente Fundación (HUSVF) under Acta número (Code) 01-2023, dated January 16, 2023, and by the Research Committee of the Internal Medicine Department de la Universidad de Antioquia. The study adhered to the provisions outlined in Resolution No. 008430 of 1993 of the Colombian Ministry of Health and the ethical principles stated in the 2013 Declaration of Helsinki. No animal research was conducted as part of this study.

Informed consent

Given the retrospective nature of the study and the use of anonymized data, the requirement for informed consent was waived by the ethics committee.

## Results

Participants

**Figure 1:**
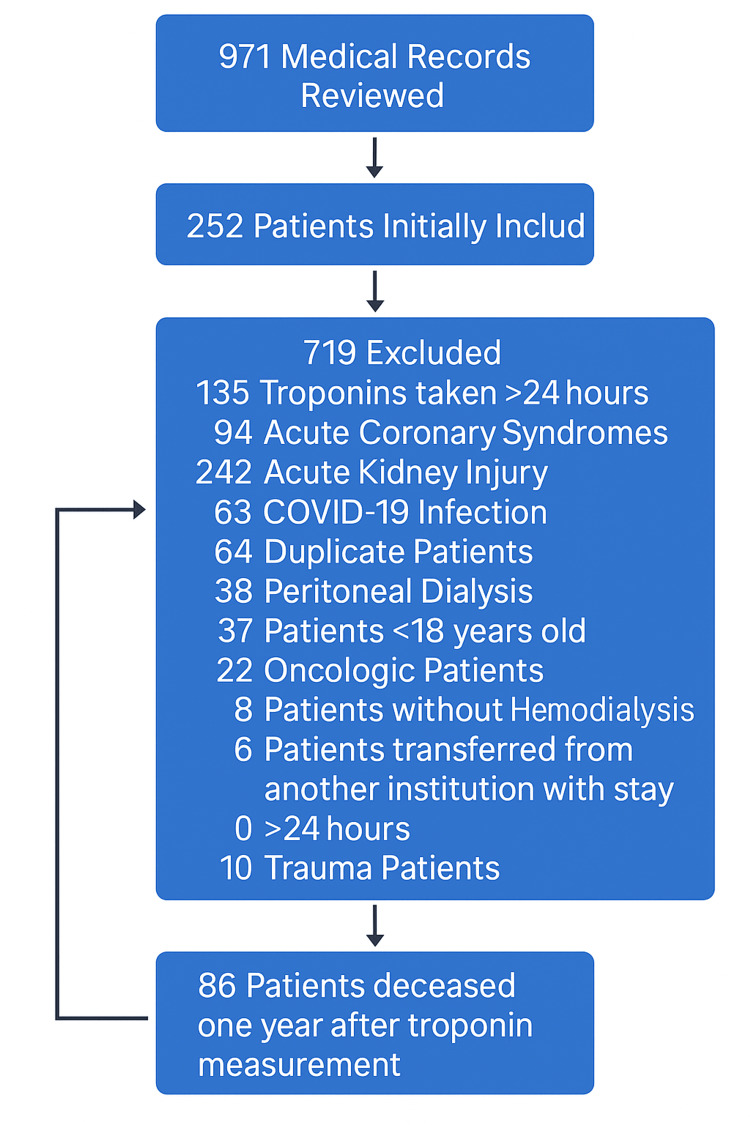
Flow diagram of patient selection and exclusions

A total of 971 medical records were screened, and 735 patients were excluded. The most common reasons for exclusion were the presence of acute kidney injury or hs-cTnI measurement performed more than 24 hours after admission to the emergency department. Ultimately, 236 patients met the eligibility criteria and were included in the final analysis (Figure 1).

Participant characteristics

A total of 236 patients were included in the study, of whom 121 (51.3%) were women and 115 (48.7%) were men. The mean age was 54.8 years. The most common etiologies of CKD were type 2 diabetes mellitus and hypertension, with hypertension being the most prevalent comorbidity, present in 228 (96.6%) patients, followed by heart failure in 120 (50.8%). The leading cause of hospitalization was acute heart failure (Appendix A). Regarding antihypertensive medication use, calcium channel blockers were the most frequently prescribed agents, used by 159 (67.3%) patients, followed by beta-blockers in 164 (64.5%) (Table 1).

**Table 1:** Descriptive data of hospitalized ESKD patients Hemoglobin, C-reactive protein, and time on dialysis are presented as mean ± SD. ACEIs, angiotensin-converting enzyme inhibitors; ARBs, angiotensin II receptor blockers; CRP, C-reactive protein; ESKD, end-stage kidney disease

Category	Total, n (%)	Alive, n (%)	Deceased, n (%)
Sex			
Male	115 (48.7%)	68 (59.1%)	47 (40.9%)
Female	121 (51.3%)	82 (67.8%)	39 (32.2%)
Mean age (years)	54.8	51.9	59.8
Troponin (Siemens)	108 (45.8%)	67 (28.3%)	41 (17.3%)
Positive (Siemens)	48 (44.4%)	26 (54.2%)	22 (45.8%)
Negative (Siemens)	60 (55.6%)	41 (68.3%)	19 (31.7%)
Troponin (Abbott)	128 (54.2%)	83 (35.1%)	45 (19.1%)
Positive (Abbott)	85 (66.4%)	49 (57.6%)	36 (42.4%)
Negative (Abbott)	43 (33.6%)	34 (79.1%)	9 (20.9%)
CRP (mg/dL)	8.26 ± 10.5	6.85 ± 8.94	10.5 ± 12.4
Hemoglobin (g/dL)	10.9 ± 2.4	11.09 ± 2.4	10.7 ± 2.5
Time on dialysis (years)	4.09 ± 4.1	3.9 ± 4.1	4.3 ± 4.0
Readmission within 3 months	76 (32.2%)	48 (20.3%)	28 (11.8%)
Etiology			
Type 2 diabetes mellitus	70 (29.6%)	33 (13.9%)	37 (15.7%)
Unknown	57 (24.1%)	41 (17.3%)	16 (6.8%)
Hypertension (etiology)	46 (19.4%)	34 (14.4%)	12 (5.0%)
Comorbidities			
Hypertension	228 (96.6%)	145 (63.6%)	83 (36.4%)
Heart failure	120 (50.8%)	68 (56.7%)	52 (43.3%)
Diabetes mellitus	93 (39.4%)	50 (53.8%)	43 (46.2%)
Smoking	63 (26.7%)	34 (54.0%)	29 (46.0%)
Acute myocardial infarction	51 (21.6%)	32 (62.7%)	19 (37.3%)
Cerebrovascular disease	24 (10.2%)	5 (20.8%)	19 (79.2%)
Antihypertensive medication use			
Calcium channel blockers	159 (67.3%)	99 (62.3%)	60 (37.7%)
Beta-blockers	164 (64.5%)	108 (65.9%)	56 (34.1%)
ACEIs/ARBs	150 (63.5%)	93 (62.0%)	57 (38.0%)

Follow-up

During the 12-month follow-up period, all-cause mortality was observed in 86 patients after the measurement of troponin I.

Primary outcome

Among the 236 patients, 133 (56.4%) had elevated hs-cTnI levels (above P99), while 103 (43.6%) had non-elevated levels. One-year all-cause mortality was higher in patients with elevated hs-cTnI, occurring in 58 (43.6%) patients, compared to 28 (27.1%) patients with non-elevated levels (Table 2).

**Table 2:** Association of Hs-cTnI with all-cause mortality, cardiovascular mortality, and readmission according to elevation above P99 Hs-cTnI, high-sensitivity cardiac troponin I; P99, 99th percentile Readmission within the first three months after Hs-cTnI measurement.

	Hs-cTnI > P99	Hs-cTnI < P99	Total
All-cause mortality	58 (44%)	28 (27.1%)	86
Cardiovascular mortality	29 (69%)	13 (31%)	42
Readmission	42 (55.2%)	34 (45%)	76

In the unadjusted analysis, elevated hs-cTnI was associated with a twofold increased risk of all-cause mortality at one year (odds ratio [OR] = 2.02; 95% confidence interval [CI], 1.19-3.60). After adjusting for age, smoking history, diabetes mellitus, and chronic heart failure, the association remained but was not statistically significant (adjusted OR = 1.73; 95% CI, 0.97-3.08) (Table 3).

**Table 3:** Multivariate analysis of the primary outcome: all-cause mortality Hs-cTnI, high-sensitivity cardiac troponin I; P99, 99th percentile Multivariate analysis adjusted for age, smoking history, diabetes mellitus, and heart failure.

Variable	OR	95% CI	p-value
Hs-cTnI > P99	1.73	0.97–3.08	0.061
Age	1.024	1.004–1.045	0.019
History of smoking	1.28	0.68–2.41	0.433
Diabetes mellitus	1.27	0.68–2.35	0.433
Heart failure	1.34	0.74–2.40	0.323

Secondary outcomes

No significant associations were observed between elevated hs-cTnI and rehospitalization within three months (adjusted OR = 0.91; 95% CI, 0.51-1.62) or one-year cardiovascular mortality (adjusted OR = 1.62; 95% CI, 0.77-3.42) (Tables 4, 5).

**Table 4:** Multivariate analysis of the secondary outcome: cardiovascular mortality Hs-cTnI, high-sensitivity cardiac troponin I; P99, 99th percentile Multivariate analysis adjusted for age, smoking history, diabetes mellitus, and heart failure.

Variable	OR	95% CI	p-value
Hs-cTnI > P99	1.62	0.77–3.42	0.198
Age	1.02	0.99–1.05	0.056
History of Smoking	1.59	0.76–3.32	0.213
Diabetes Mellitus	0.66	0.30–1.43	0.295
Heart Failure	2.07	0.97–4.42	0.058

**Table 5:** Multivariate analysis of the outcome: readmission within three months Hs-cTnI, high-sensitivity cardiac troponin I; P99, 99th percentile Multivariate analysis adjusted for age, smoking history, diabetes mellitus, and heart failure.

Variable	OR	95% CI	p-value
Hs-cTnI > P99	0.91	0.51–1.62	0.769
Age	0.99	0.92–1.01	0.434
History of smoking	0.81	0.42–1.56	0.530
Diabetes mellitus	1,03	0.55–1.98	0.906
Heart failure	1.97	1.08–3.57	0.025

## Discussion

In this retrospective cohort of patients with ESKD on HD who presented to a high-complexity emergency department without symptoms of ACS, we found no statistically significant association between elevated hs-cTnI levels (above P99) and all-cause or cardiovascular mortality at 12 months nor with three-month rehospitalization. Although a numerical trend was observed, with higher absolute and relative frequencies of mortality among patients with elevated hs-cTnI, the confidence intervals suggest the possibility of random error.

These findings indicate that hs-cTnI may not serve as an independent prognostic biomarker in this population, despite the elevated levels commonly observed in HD patients. This lack of association persisted even after adjusting for key confounders, including comorbidities, cardiovascular risk factors, medications, and hospitalization causes.

Previous studies have reported mixed results regarding the prognostic utility of hs-cTnI in ESKD. Snaedal et al. found that high high-sensitivity cardiac troponin T (hs-cTnT), but not hs-cTnI, was associated with cardiovascular mortality, suggesting that hs-cTnT may be more suitable for risk stratification in ESKD due to its longer half-life, greater biochemical stability, lower renal clearance, and reduced binding to dialysis membranes [17]. Similarly, Hickman et al. reported that both hs-cTnT and hs-cTnI predicted five-year mortality in HD patients, but only hs-cTnT remained statistically significant after adjusting for confounders [18].

Consistent with our results, both Snaedal et al. and Hickman et al. found that the association between hs-cTnI and mortality was attenuated after multivariable adjustment, including for variables such as albumin, C-reactive protein, dialysis modality, and history of vascular disease [17-18]. These findings support the hypothesis that elevated hs-cTnI in HD patients may reflect chronic inflammation, volume overload, or subclinical myocardial injury, rather than serving as a direct predictor of adverse outcomes.

In contrast, other studies have suggested a prognostic role for hs-cTnI. Sandoval et al. observed significant associations between hs-cTnI and both all-cause mortality and sudden death in HD outpatients followed with serial measurements over three months [19]. However, their analysis adjusted only for age, sex, and race. Likewise, Maresca et al. reported an association between hs-cTnI and cardiovascular mortality at 12 months (p < 0.001), though the study involved a small sample and limited adjustment for confounding (age and prior heart disease) [16].

The more comprehensive adjustment in our study may explain the divergence in findings, as previously observed associations could be due in part to residual confounding. Moreover, longer follow-up durations in other studies (up to five years) may have allowed detection of delayed mortality outcomes. A longer follow-up in our cohort could have altered the observed associations, particularly if hs-cTnI elevation reflects long-term rather than short-term risk.

Taken together, our findings suggest that hs-cTnI, when assessed at a single time point in asymptomatic HD patients, may not provide reliable prognostic information regarding mortality. This underscores the need for more comprehensive approaches to risk stratification. Future studies should evaluate serial hs-cTn measurements and explore the utility of alternative biomarkers, such as hs-cTnT, NT-proBNP, or inflammatory markers, to improve risk prediction in this population. Prospective studies with standardized assays and robust confounding control are warranted to clarify the role of hs-cTnI in ESKD prognosis.

Limitations

This study has some limitations. The small sample size may have reduced statistical power to detect significant associations. A change in the troponin assay during the study period limited analysis to a dichotomous variable, potentially decreasing sensitivity to risk gradations. The one-year follow-up may have been insufficient to capture long-term outcomes. Additionally, as the study was conducted in a tertiary hospital, selection bias is possible due to the higher comorbidity burden in this population. Lastly, the retrospective design limits control over residual confounding.

## Conclusions

Although mortality was higher among patients with elevated hs-cTnI (43.6%) compared to those with non-elevated levels (27.1%), no statistically significant association between hs-cTnI elevation and mortality was found after adjustment for confounding factors. These results suggest that hs-cTnI may not be an independent prognostic biomarker in HD patients without ACS symptoms. Prospective studies comparing the prognostic performance of hs-cTnI and hs-cTnT, including serial measurements and comprehensive confounder adjustment, are needed to better define their roles in mortality risk prediction in ESKD patients on hemodialysis.

## Appendices

Appendix A. Definition of Cardiovascular Death

Cardiovascular death is defined as death caused by cardiovascular mechanisms. This includes death due to acute myocardial infarction (AMI), which refers to death occurring within 30 days after an AMI or as an immediate consequence of it, such as heart failure or refractory arrhythmia. It also encompasses complications related to AMI treatment, including percutaneous coronary intervention, coronary artery bypass grafting, or hospitalization during the infarction. Sudden death is defined as witnessed death occurring without new symptoms, witnessed death occurring within 60 minutes after the onset of cardiac symptoms unless those symptoms suggest AMI, witnessed death attributable to an identified arrhythmia (documented on ECG, monitor, or implantable cardioverter-defibrillator), or unwitnessed death in a patient last seen alive and clinically stable within 72 hours before being found dead without evidence of a non-cardiovascular cause. Death due to heart failure includes deaths associated with clinical worsening of heart failure symptoms or signs, regardless of its etiology, whether ischemic or non-ischemic heart disease, hypertension, or other causes. Death due to ischemic stroke, complications of cardiac procedures, and cardiovascular hemorrhage, such as intracranial hemorrhage, ruptured aortic aneurysm, and hemorrhagic cardiac tamponade, are also included in the definition of cardiovascular death.

**Table 6:** Etiologies of end-stage kidney disease, causes of hospitalization, and use of antihypertensive medications ANCA-MPO, anti-neutrophil cytoplasmic antibody – myeloperoxidase *Group of less frequent causes that did not meet the threshold for individual reporting. These include gastrointestinal bleeding, infections, seizures, electrolyte imbalances, and other non-cardiovascular complications.

		Total	Alive	Deceased
Etiology	Type 2 diabetes mellitus	70	33	37
	Unknown	57	41	16
	Hypertension	46	34	12
	Systemic lupus erythematosus	14	11	3
	Obstructive disease	11	4	9
	Focal segmental glomerulosclerosis	7	5	2
	Type 1 diabetes mellitus	6	4	2
	IgA nephropathy	5	4	1
	Polycystic kidney disease	5	3	2
	Cocaine-induced vasculitis	4	3	1
	ANCA-MPO vasculitis	3	2	1
	Thrombotic microangiopathy	2	2	0
	NSAID-induced nephropathy	2	1	1
	Membranoproliferative glomerulonephritis	1	1	0
	Amyloidosis	1	0	1
	HIV-associated nephropathy	1	0	1
	Tubulointerstitial nephritis	1	1	0
Use of antihypertensive medications	Calcium channel blockers	159	99	60
	Beta-blockers	164	108	56
	Angiotensin-converting enzyme inhibitors/angiotensin II receptor blockers	150	93	57
	Alpha-blockers	90	54	36
	Alpha agonists	77	48	29
	Diuretics	51	36	15
	Minoxidil	42	31	11
	Mineralocorticoid receptor antagonists	6	6	0
Causes of admission	Acute heart failure	109	64	45
	Hypertensive crisis	91	61	30
	Atrial fibrillation with rapid ventricular response	15	10	5
	Stroke	6	5	1
	Pulmonary thromboembolism	3	3	0
	Myopericarditis	2	2	0
	Other*	122		
